# Crucial Role of Reactive Oxygen Species (ROS) for the Proapoptotic Effects of Indirubin Derivatives in Cutaneous SCC Cells

**DOI:** 10.3390/antiox10101514

**Published:** 2021-09-24

**Authors:** Jiaqi Zhu, Peter Langer, Claas Ulrich, Jürgen Eberle

**Affiliations:** 1 Skin Cancer Center, Department of Dermatology, Venerology and Allergology, Charité Universitätsmedizin Berlin, 10117 Berlin, Germany; zhujiaqijiaqi@gmail.com (J.Z.); claas.ulrich@charite.de (C.U.); 2 Department of Gynecology and Obstetrics, Jilin University, Changchun 130000, China; 3 Institute of Chemistry, University of Rostock, 18051 Rostock, Germany; peter.langer@uni-rostock.de; 4 Leibniz Institute of Catalysis, University of Rostock, 18051 Rostock, Germany

**Keywords:** cutaneous SCC, indirubin, apoptosis, reactive oxygen species (ROS), antioxidants

## Abstract

Efficient drugs are needed for countering the worldwide high incidence of cutaneous squamous cell carcinoma (cSCC) and actinic keratosis. Indirubin derivatives represent promising candidates, but their effects in cSCC cells have not been reported before. Here, we investigated the efficacy of three indirubin derivatives (DKP-071, -073 and -184) in four cSCC cell lines. High efficacy was seen in SCL-I, SCL-II, SCC-12 and SCC-13, resulting in up to 80% loss of cell proliferation, 60% loss of cell viability and 30% induced apoptosis (10 µM). Apoptosis was further enhanced in combinations with TNF-related apoptosis-inducing ligand (TRAIL). Induction of reactive oxygen species (ROS) appeared as critical for these effects. Thus, antioxidative pretreatment completely abolished apoptosis as well as restored cell proliferation and viability. Concerning the pathways, complete activation of caspases cascades (caspases-3, -4, -6, -7, -8 and -9), loss of mitochondrial membrane potential, activation of proapoptotic PKCδ (protein kinase C delta), inhibition of STAT3 (signal transducer and activator of transcription 3), downregulation of antiapoptotic XIAP (X-linked inhibitor of apoptosis protein) and survivin as well as upregulation of the proapoptotic Bcl-2 protein Puma and the cell cycle inhibitor p21 were obtained. Importantly, all activation steps were prevented by antioxidants, thus proving ROS as a master regulator of indirubins’ antitumor effects. ROS induction presently develops as an important issue in anticancer therapy.

## 1. Introduction

Actinic keratosis (AK) derives from neoplastic epidermal keratinocytes and is characterized by high prevalence and the risk to proceed into invasive cutaneous squamous cell carcinoma (cSCC). Cutaneous SCC accounts for about 20% of skin malignancies and about 20% of skin cancer deaths worldwide [1,2]. For Caucasians and East Asian populations (including Japanese and Chinese), cSCC is the second most commonly diagnosed skin cancer, following basal cell carcinoma [3,4], whereas for black people, it is the most commonly diagnosed skin cancer [5]. New and alternative therapeutic options are needed for early and late disease.

The elimination of tumor cells by the induction of apoptosis represents a principal goal in cancer therapy, while therapy resistance is frequently explained by apoptosis deficiency [6]. Thus, resistance to apoptosis represents a crucial step in oncogenesis and drug resistance [7]. Three mayor types of cell death have been distinguished, namely type I (apoptosis), type II (autophagy) and type III (necrosis) [8]. Intrinsic proapoptotic pathways can be activated in response to cellular stress situations, including high levels of reactive oxygen species (ROS) as well as by anticancer treatment, e.g., by chemotherapy. This relies on mitochondrial outer membrane permeability (MOMP), loss of mitochondrial membrane potential and release of mitochondrial factors such as cytochrome c, which may trigger activation of initiator caspase-9 [9]. This step is critically controlled by the family of pro- and anti-apoptotic Bcl-2 proteins. Thus, Bcl-2 exerts its antiapoptotic function through binding and inhibition of the proapoptotic family member Bax [8,9]. Often, activation of apoptosis and inhibition of cell proliferation are regulated in parallel. Thus, the cyclin-dependent kinase inhibitor p21 (Cip1/Waf1) represents a major target of p53, which also drives the expression of several proapoptotic factors [10].

On the other hand, extrinsic induction of apoptosis is initiated by death ligands such as CD95L/FasL and TRAIL (TNF-related apoptosis-inducing ligand). Upon death receptor activation, cell membrane-bound death-inducing signaling complexes are formed, resulting in activation of initiator caspases, such as caspase-8 and caspase-10 [11]. In particular, the death ligand TRAIL has attracted much consideration due to its anticancer activity, while normal cells are largely spared [12,13]. Furthermore, caspase-4 may be involved in endoplasmic reticulum stress-induced apoptosis, besides its roles in inflammation [14,15]. Initiator caspases may cleave and thus activate effector caspases such as caspases-3, -6 and -7, which themselves cleave a large number of death substrates with the final result of DNA fragmentation and apoptosis induction [16].

Additionally, several signaling pathways contribute to the regulation of apoptosis. Thus, the STAT3 pathway can be activated in response to cytokines and growth factors. STAT3 then translocates to the cell nucleus and acts as a transcription activator for a variety of genes that mediate cell growth and inhibition of apoptosis [17]. The protein kinase C (PKC) family of isoenzymes encloses several serine-threonine kinases, which are involved in the regulation of different cellular processes, including cell proliferation, cell differentiation and apoptosis [18]. While PKCα and PKCβ in particular support cell proliferation and cell invasion [19], PKCδ was reported as proapoptotic. Following phosphorylation and translocation steps, PKCδ can be activated through processing, which releases the active catalytic domain (41 kDa) from its 78 kDa proform. Activated PKCδ was related to the induction of apoptosis through tyrosine phosphorylation and thus activation of caspase-3 [20,21].

Reactive oxygen species (ROS) are involved in several signaling pathways. The formation of different kinds of ROS can result in molecular damage and increased oxidative activity. Thus, ROS play important roles in different kinds of diseases of the neuronal, cardiovascular and nervous systems, as well as in aging [22]. Furthermore, ROS may contribute to the regulation of apoptosis, as shown in melanoma cells by an iron-substituted nucleoside analogue [23], in cSCC cells for celecoxib [24] and in cutaneous T-cell lymphoma cells (CTCL) for an indirubin derivative [25]. ROS may derive from mitochondrial leakage or other sources [26], but their relation to the described apoptosis pathways is less clear to date.

Indirubin has been identified as the active ingredient of a traditional Chinese herbal medicine (Danggui Longhui Wan), used for treatment of chronic and inflammatory diseases. Clinical results from the 1980s, which have been obtained in chronic myelocytic leukemia patients treated with indirubin, stimulated several studies on this compound [27,28]. Due to the only limited antitumor activity of the native form, the structure of indirubin is presently employed in several laboratories as a skeleton for the synthesis of new derivatives to increase its antitumor effects [29,30,31].

We have previously reported the synthesis of a new series of indirubin derivatives based on N-glycosylated 3-alkylideneoxindoles containing halogen substituents [32]. Here, we investigated the proapoptotic effects of DKP-071, DKP-073 and DKP-184 in cSCC cells and unraveled the downstream signaling pathways. These appeared as essentially based on the production of reactive oxygen species, which opens new perceptions in the proapoptotic targeting of cSCC cells.

## 2. Materials and Methods

### 2.1. Cell Culture and Treatment

For investigating the effects of indirubin derivatives DKP-071, DKP-073 and DKP-184, we investigated four cSCC cell lines (SCL-I, SCL-II, SCC-12 and SCC-13). There is not much information in the literature to distinguish these cell lines by their origin, as all four derive from human facial skin. Only in terms of sensitivity, we found in previous studies that SCL-I is resistant to diclofenac/hyaluronic acid, while others were sensitive [33]. The HaCaT cell line was reported to derive from spontaneously immortalized keratinocytes [34]. Cells were maintained at 5% CO_2_ in RPMI 1640 growth medium (Life Technologies, Darmstadt, Germany) supplemented with 10% FCS, 2 mM glutamine and non-essential amino acids. Most assays were performed in 24-well plates, and 4 × 10^4^ cells were seeded per well.

Cells were treated with KillerTRAIL™ (Adipogen, San Diego, CA, USA; AG-40T-0001; 50 ng/mL) and with the indirubin derivatives DKP-071, DKP-073 and DKP-184 (2.5–20 μM) [32], whereas control cells received only the solvent DMSO. Cells were also treated with non-substituted indirubin (BioMol, Hamburg, Germany; TGM-T6169) at equimolar concentrations. For caspase inhibition, cells were treated with the pan-caspase inhibitor QVD-Oph (Abcam, Cambridge, UK; 10 μM), which was applied 1 h before cells were treated with agonists. For inhibition of PKCδ, bisindolylmaleimide I (Cayman Chemical, Ann Arbor, MI, USA) was used at 1 µM.

### 2.2. Cell Proliferation Assays

Cell proliferation was determined by WST-1 assay (Roche Diagnostics, Penzberg, Germany), which depends on the cleavage of the water-soluble tetrazolium salt by mitochondrial dehydrogenases in metabolically active cells. WST-1 detects live cells and can thus by used for counting only viable cells. The read-out is thus complex and reflects both cell number and viability of quantified cells.

### 2.3. Determination of Apoptosis, Cell Viability and Cytotoxicity

Quantification of apoptosis was performed by cell cycle analysis. Cells were harvested by trypsinization, and all cells were lysed in hypotonic buffer. In this way, cells’ nuclei were isolated and were further stained for 1 h with 40 mg/mL of propidium iodide (Sigma-Aldrich, St. Louis, MO, USA). Cells in G1, G2 and S-phase, as well as sub-G1 cells, were depicted by flow cytometry at FL3A using a FACS Calibur (BD Bioscience, Bedford, MA, USA). Due to the washing out of small DNA fragments, nuclei with less DNA than G1 (sub-G1) correspond to apoptotic cells. Thus, the increase of the sub-G1 fraction indicates the percentage of cells with DNA fragmentation (apoptotic cells).

Cell viability was determined by staining cells with calcein-AM (PromoCell, Heidelberg, Germany), which is converted in viable cells by intracellular esterases to green fluorescent calcein. Cells, grown and treated in 24-well plates, were harvested by trypsinization and stained with 2.5 µg/mL of calcein-AM at 37 °C for 1 h. Labeled cells were washed with PBS and measured by flow cytometry (FL2H).

Possible cytotoxic effects were determined at 4 and at 24 h of treatment by quantification of lactate dehydrogenase (LDH) activity in cell culture supernatants. Released LDH is indicative for damaged and cytotoxic cells. LDH activity was determined in an ELISA reader after applying a WST-1 cytotoxicity detection assay (Roche Diagnostics, Penzberg, Germany).

### 2.4. Mitochondrial Membrane Potential

Mitochondrial membrane potential (Δψm) was determined by staining cells with the fluorescent dye TMRM^+^ (Sigma-Aldrich, Darmstadt, Germany). Cells, grown and treated in 24-well plates, were harvested by trypsinization and stained for 20 min at 37 °C with 1 µM of TMRM^+^. After 2-times washing with PBS, cells were measured by flow cytometry (FL2H).

### 2.5. Analysis of Reactive Oxygen Species (ROS)

For determination of intracellular ROS levels, cells grown in 24-well plates were pre-incubated for 1 h with the fluorescent dye H_2_DCF-DA (D-399, Thermo Fisher Scientific, Hennigsdorf, Germany, 10 µM), before starting treatment with effectors. After 2–24 h of treatment, cells were harvested by trypsinization, washed several times with PBS and analyzed by flow cytometry (FL1H). As a positive control, treatment with H_2_O_2_ (1 mM, 1 h) was applied. Antioxidative treatments were used, aiming at the suppression of indirubin-induced ROS levels. Thus, N-acetylcysteine (NAC, Sigma-Aldrich, Taufkirchen, Germany) was used in concentrations of up to 1 mM and was generally applied 1 h before starting indirubin treatments.

### 2.6. Western Blotting

For Western blotting, total protein extracts were obtained by a cell lysis buffer containing 150 mM NaCl, EDTA (1 mM), 1% NP-40, 50 mM Tris (pH 8.0) as well as phosphatase and protease inhibitors. Following SDS polyacrylamide gel electrophoresis, proteins were blotted on nitrocellulose membranes.

Primary antibodies of Cell Signaling (Danvers, MA, USA) were: Caspase-3 (9662, rabbit, 1:1000), Cleaved caspase-3 (9664, rabbit, 1:1000), Caspase-8 (9746, mouse, 1:1000), Caspase-9 (9502, rabbit, 1:1000), Caspase-6 (9762, rabbit, 1:1000), Caspase-7 (9492, rabbit, 1:1000), XIAP (2042, rabbit, 1:1000), Mcl-1 (4572, rabbit, 1:1000), Bad (9292, rabbit, 1:1000), Bcl-w (2724, rabbit, 1:1000), Bcl-2 (2872, rabbit, 1:1000). Primary antibodies of Santa Cruz Biotech (Dallas, TX, USA) were: Caspase-4 (sc-1229, goat, 1:500), c-FILP (sc-5276, mouse, 1:500), survivin (sc-177779, mouse, 1:500), p21(sc-6246, mouse, 1:500), β-actin (sc-47778, mouse, 1:1000), Puma (sc-374223, mouse, 1:500), Bax (sc-7480, mouse, 1:500), Bak (sc-832, mouse, 1:500). Primary antibody of Abcam (Cambridge, UK) was: DR5 (ab8416, rabbit, 1:1000). Secondary antibodies were: peroxidase-labeled goat anti-rabbit and goat anti-mouse (Dako, Hamburg, Germany; 1:5000).

### 2.7. Statistical Analyses

Each finding was proven by at least two independent experiments. For all assays, each independent experiment itself consisted of at least three independent values (three individual wells that were seeded, treated and analyzed individually). Thus, we had at least six values in one group, which were used for statistical analysis. Statistical significance was determined by Student’s t-test and is indicated by asterisks in the figures (* *p* < 0.05; ** *p* < 0.01; *** *p* < 0.001).

For semi-quantitative protein analysis, Western blot signals were quantified by densitometry using Fusion-Capt Advance software (Vilber Lourmat, Collégien, France), values were normalized by the respective β-actin values and median values were formed from each two independent experiments (independent cell extracts).

## 3. Results

### 3.1. Decreased cSCC Cell Proliferation, Induction of Apoptosis and Loss of Cell Viability

**Figure 1:**
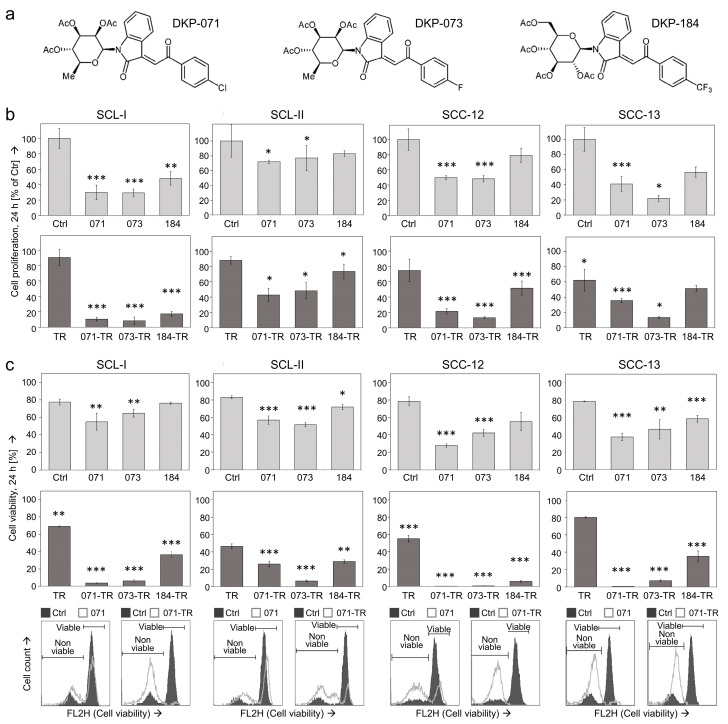

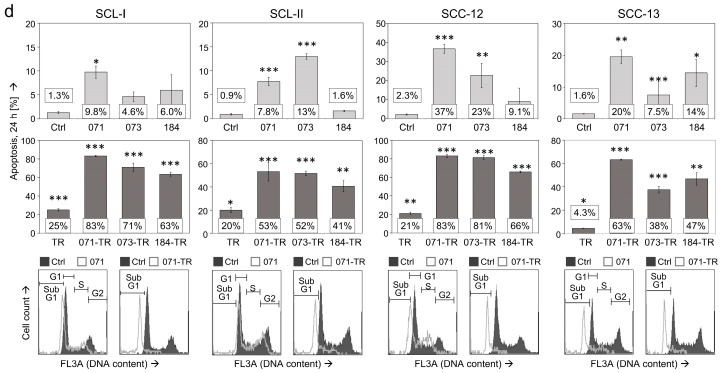
Decreased cell proliferation, cell viability and apoptosis induction. (**a**) Chemical structures of indirubin derivatives DKP-071, DKP-073 and DKP-184 are shown. (**b**) Cell proliferation was quantified by WST-1 assay in four cSCC cell lines at 24 h of treatment with indirubins (DKP-071, -073 and -184, 10 μM), TRAIL (TR; 50 ng/mL) or the combinations, as indicated. (**c**) Cell viability was determined by calcein-AM staining in cSCC cell lines at 24 h (treatment as above). (**c**) Apoptotic cells characterized by DNA fragmentation were identified by flow cytometry after propidium iodide staining as sub-G1 cells (cell cycle analyses, treatment as above). (**b**–**d**) Mean values are shown of triplicates of representative experiments (at least two independent experiments, each one with triplicates). (**c**,**d**) Examples of flow cytometry measurements are provided below (overlays of treated cells vs. controls). (**d**) The percentages of apoptotic cells are also indicated. Asterisks indicate statistical significance, as compared to controls (* *p* < 0.05; ** *p* < 0.01; *** *p* < 0.001).

As indirubin and its derivatives represent promising candidates for cSCC therapy, we investigated the effects of three indirubin derivatives (DKP-071, -073, -184; Figure 1a) in four representative cSCC cell lines (SCL-I, SCL-II, SCC-12 and SCC-13). As the death ligand TRAIL is expressed by immune cells in an antitumor immune response and as TRAIL agonists were already tested in clinical trials, combinations of the three indirubins with TRAIL were also investigated.

As cell proliferation represents an important issue in anticancer treatment, it was monitored by quantitative WST-1 assays, which determine live cells, in cSCC cell lines treated with 10 μM indirubin ± 50 ng/mL TRAIL. Cell proliferation rates at 24 h of treatment were generally reduced in cSCC cells by DKP-071, -073 and -184, reaching values of 30%, 29% and 48% (SCL-I), of 72%, 77% and 83% (SCL-II), of 50%, 48% and 79% (SCC-12) as well as of 41%, 22% and 57% (SCC-13) (Figure 1b). These antiproliferative effects were further strongly enhanced by the combination of indirubins with TRAIL (Figure 1b).

Even more pronounced combination effects were seen at the level of cell viability, as determined by calcein staining and flow cytometry. At 24 h, cell viability was almost completely abolished by the combinations of DKP-071 or DKP-073 with TRAIL (<10%), whereas cell viability after single treatments with DKP-071 and DKP-073 ranged between 35% and 80%. The effects of DKP-184 were generally somewhat less pronounced (Figure 1c).

Some induction of apoptosis was also seen at 24 h for DKP-071, -073 and -184 when applied alone, resulting in rates of 10%, 5% and 6% (SCL-I), 8%, 13% and 2% (SCL-II), 37%, 23% and 9% (SCC-12) as well as 19%, 8% and 14% (SCC-13) (Figure 1d). Most striking, however, was the enhancement of apoptosis by the combination of indirubins and TRAIL, resulting in apoptosis rates of 84%, 70% and 63% (SCL-I), 53%, 51% and 40% (SCL-II), 83%, 81% and 65% (SCC-12) and 63%, 38% and 47% (SCC-13) (Figure 1d). Of note, in response to indirubin treatments, the whole cell populations analyzed by flow cytometry were responsive and shifted in the direction of the sub-G1 area. Thus, the same marker for sub-G1 cell populations was used as in the controls (Figure 1d, insets). The high apoptosis values were approved by the particularly strong caspase activation, seen after combination treatments in Western blots (shown below).

The effects on apoptosis and cell viability were not associated with cytotoxicity, as determined by quantification of release of lactate dehydrogenase (LDH). There was no indication of increased cell necrosis in terms of LDH release, after treatment with the three indirubin derivatives as well as after combination treatments with TRAIL. This was found both at 4 h, excluding direct cytotoxic effects of the substances, as well as at 24 h, also largely excluding secondary necrotic effects (Supplementary Figure S1a,b).

The effects of indirubin derivatives were much stronger than those of non-substituted indirubin. This was shown in SCL-I and SCC-12 by applying equimolar concentrations (5, 10, 20 µM) of DKP-071 and non-substituted indirubin. While DKP-071 significantly triggered apoptosis and loss of cell viability when applied in concentrations of 10 and 20 µM, the effects of non-substituted indirubin were much less pronounced and resulted in only less than 5% apoptosis and at maximum in a reduction of cell viability to 67%, when applied at 20 µM (Supplementary Figure S2a,b). However, the effects of indirubin derivatives appeared as not absolutely tumor-specific, as also some response was seen in HaCaT immortalized keratinocytes (up to 13% apoptosis, decrease of cell viability to 33%, Supplementary Figure S3). Thus, the combination of indirubin derivatives and TRAIL appeared as a promising strategy for targeting of cSCC cells.

### 3.2. Changes of Mitochondrial Membrane Potential

**Figure 2:**
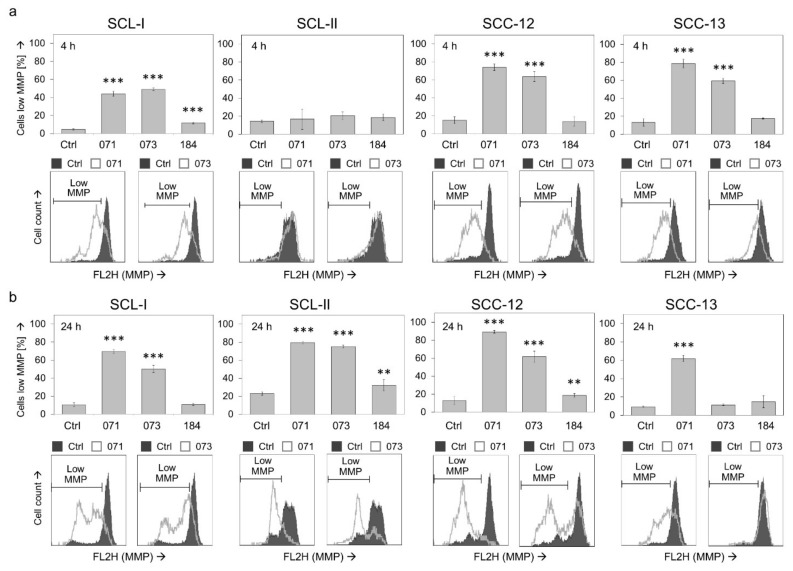
Loss of mitochondrial membrane potential (MMP). Percentages of cells with low MMP were determined by TMRM^+^ staining at 4 h (**a**) and at 24 h (**b**) of treatment with indirubins (DKP-071, -073 and -184, 10 μM) in four cSCC cell lines. Cells with low MMP are defined by less staining than the main peak of control cells. Example flow cytometry measurements of controls and treatments are provided below (overlays of treated cells vs. controls), and cell fractions with low MMP are indicated. Mean values and SDs are shown of a representative experiment (two independent experiments, each one with triplicates). Asterisks indicate statistical significance, as compared to controls (** *p* < 0.01; *** *p* < 0.001).

Addressing the mechanisms that mediate the antitumor effects of indirubins in cSCC cells, we determined changes in mitochondrial membrane potential (MMP) in response to indirubin treatments, applying TMRM^+^ staining and flow cytometry. Loss of MMP was seen in SCL-I, SCC-12 and SCC-13 already at 4 h (44–78%, for DKP-071 and -073), indicative of an activation of mitochondrial apoptosis pathways (Figure 2a). Only SCL-II showed a delayed response, with no effects at 4 h but strong loss of MMP at 24 h (80%, 75% for DKP-071 and -073; Figure 2b). Thus, loss of MMP appeared as an early effect (4 h), at least in cell lines SCL-I, SCC-12 and SCC-13. At this time, apoptosis and loss of cell viability were not clearly evident. Thus, we can conclude that loss of MMP represents an initial effect, not secondary to cell death.

### 3.3. High ROS Production in Response to Indirubin Derivatives in cSCC Cells

**Figure 3:**
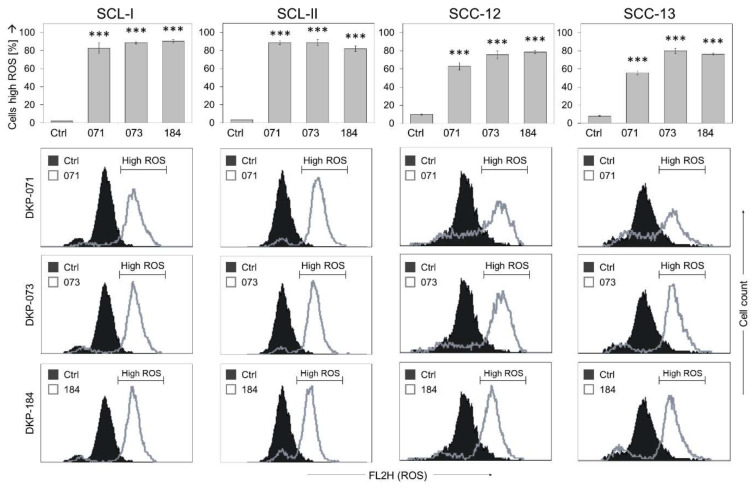
Changes in ROS production. ROS levels were determined by H_2_DCF-DA staining in four cSCC cell lines at 4 h of treatment with indirubins (DKP-071, -073 and -184, 10 μM). Example cytometry measurements of controls and treatments are provided below (overlays of treated cells vs. controls), and cell fractions with high ROS are indicated. Mean values and SDs are shown of a representative experiment (two independent experiments, each one with triplicates). Asterisks (***) indicate high statistical significance, as compared to controls (*p* < 0.001).

Increased evidence in recent years has shown that reactive oxygen species (ROS) may have vital roles in skin cancer therapy, related to induction of apoptosis. Production of ROS in response to indirubin treatment appeared as a very general and early effect in cSCC cells, as determined by H_2_DCF-DA staining and flow cytometry. Of note, the effects of indirubins on ROS levels were complete, namely the whole cell population shifted to higher ROS levels, as seen in flow cytometry (Figure 3, insets). Thus, at 4 h of indirubin treatment, the cell proportions with high ROS levels were generally between 60% and 90% (Figure 3). Increased ROS levels appeared as a most pronounced response, affected in each cell line in response to the indirubin derivatives.

We aimed to see whether ROS production correlated with the effects on cell viability and apoptosis. Thus, ROS levels were quantified in response to increasing concentrations (2.5, 5, 10 and 20 µM) of a selected indirubin (DKP-071) in two cell lines (SCL-I and SCC-12). In parallel, assays for apoptosis and cell viability were performed. The experiments revealed that strong ROS production was seen only at concentrations of 10 and 20 µM, while 2.5 and 5 µM were much less effective (Supplementary Figure S4). Similarly, significant loss of cell viability and induction of apoptosis were also seen only for these higher concentrations (Supplementary Figure S2a,b). Thus, high ROS production correlated with loss of cell viability and apoptosis induction.

ROS production was also determined at different times: 1, 2, 4 and 24 h. The main finding was that ROS comes particularly early, as shown in SCL-I and SCC-12 at 1 h (>90% cells with high ROS), and high ROS levels remained at least for 4 h (Supplementary Figure S5a), while at 24 h, percentages appeared as somewhat reduced (30–80%), possibly due to some ongoing cell proliferation (Supplementary Figure S5b).

### 3.4. Strong Caspase Activation in Course of Combined Treatment

Apoptosis can be regulated by caspase-dependent as well as by caspase-independent mechanisms. Thus, activation of the caspase cascade was investigated by Western blot analysis exemplarily in one of the 4 cell lines (SCL-I). Analyses included the effector caspases-3, -6 and -7, the initiator caspase-8 of the extrinsic pathway, the initiator caspase-9 of the intrinsic pathway as well as caspase-4, which may be involved in endoplasmic reticulum stress-induced apoptosis [14].

**Figure 4:**
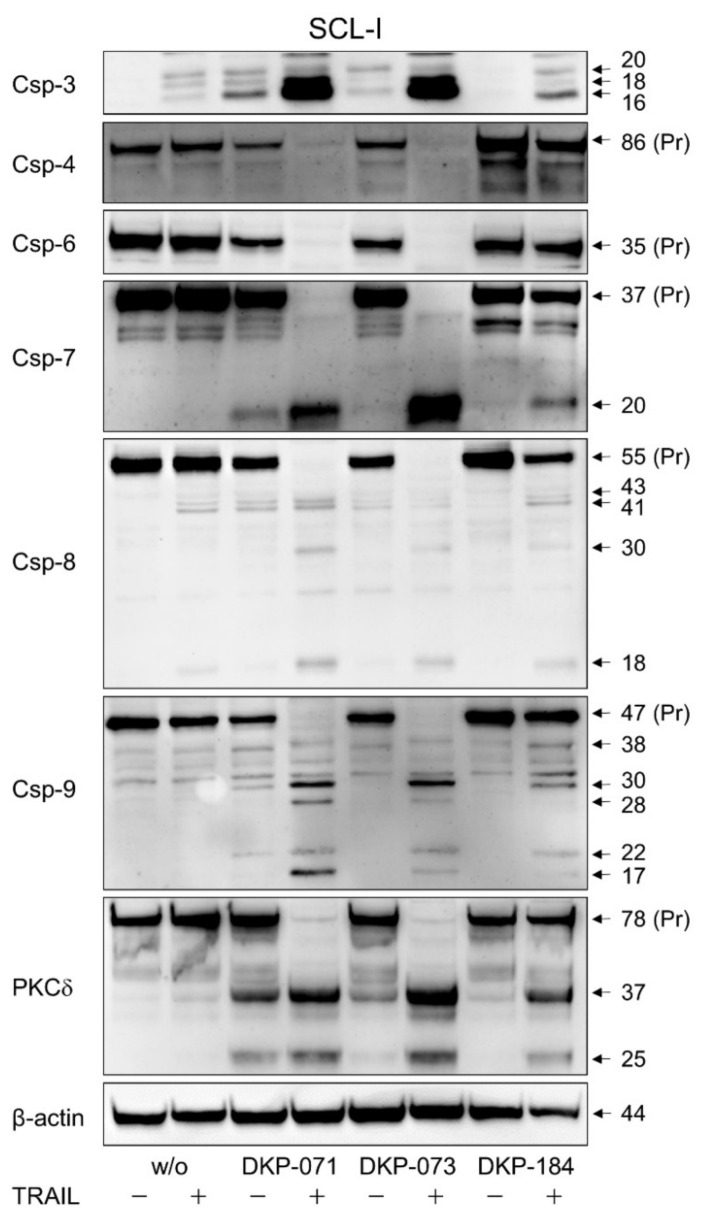
Enhanced caspase activation in course of combined treatment. Protein extracts of SCL-I cells treated for 24 h with indirubins (DKP-071, -073 and -184, 10 μM), TRAIL (50 ng/mL) or the combinations were analyzed by Western blotting and compared to control cells. Equal protein amounts (30 µg per lane) were separated by SDS-PAGE, and consistent blotting was proven by Ponceau staining as well as by evaluation of β-actin expression. Proforms (Pr) and characteristic cleavage products are indicated, such as for caspase-3 (20, 18, 16 kDa), caspase-4 (86 kDa), caspase-6 (35 kDa), caspase-7 (37, 20 kDa), caspase-8 (55, 43, 41, 18 kDa), caspase-9 (47, 38, 17 kDa) and PKCδ (78, 37, 25 kDa). Besides these, some intermediate and secondary cleavage products were seen. Two independent series of protein extracts and independent Western blots revealed highly comparable results.

Treatment with indirubins alone showed some effects on caspase processing, indicative of their activation, however, the strongest caspase activation was obtained in the combinations of indirubins with TRAIL, in agreement with the apoptotic rates shown above. Thus, the proforms of caspase-4 (86 kDa), caspase-6 (35 kDa), caspase-7 (37 kDa), caspase-8 (55 kDa) and caspase-9 (47 kDa) were almost completely degraded at 24 h in response to DKP-071/TRAIL and DKP-073/TRAIL treatment (Figure 4). In parallel, characteristic processing products were obtained, such as of caspase-3 (20, 18, 16 kDa), caspase-7 (20 kDa), caspase-8 (43, 41, 18 kDa) and caspase-9 (37, 17 kDa), as well as some other intermediate or secondary processing products (Figure 4). Densitometric, semiquantitative analyses and normalization with the β-actin signals largely confirmed this strong regulation of caspases. The caspase processing in DKP-184-treated cells was generally somewhat weaker than DKP-071 and -073 (Figure 4), also in parallel with the apoptosis values provided above. Collectively, these data indicate complete activation of caspase cascades by indirubin/TRAIL combinations.

**Figure 5:**
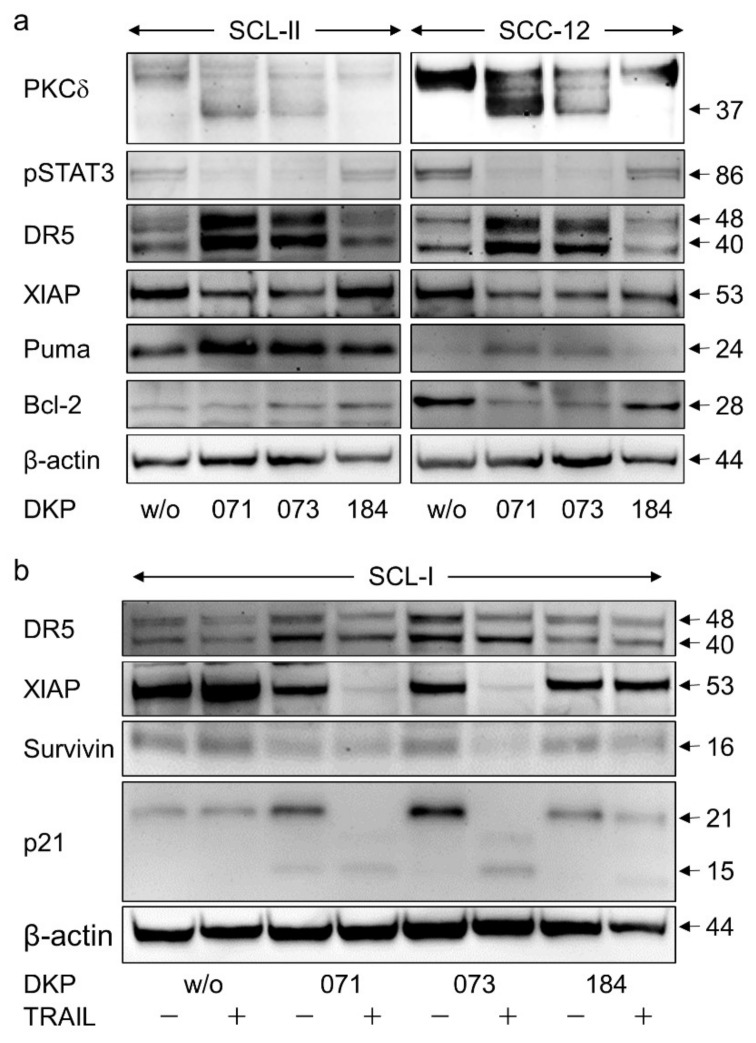
Expression of apoptosis agonists. (**a**) Cell lines SCL-II and SCC-12 were treated for 24 h with indirubins (DKP-071, -073 and -184; 10 μM). (**b**) Cell line SCL-I was treated for 24 h with indirubins (DKP-071, -073 and -184, 10 μM) ± TRAIL (50 ng/mL). (**a**,**b**) Expression of PKCδ (fragment 37 kDa), pSTAT3 (86 kDa), TRAIL-R2/DR5 (48, 40 kDa), XIAP (53 kDa), Puma (24 kDa), Bcl-2 (28 kDa), survivin (16 kDa) and p21 (21, 15 kDa) was analyzed by Western blotting. Equal protein amounts (30 µg per lane) were separated by SDS-PAGE, and consistent blotting was proven by Ponceau staining as well as by evaluation of β-actin expression. Size of proteins (in kDa) is indicated on the right side. Largely similar results were obtained in three independent Western blot experiments using three independent series of cell extracts.

Proapoptotic pathways may also be initiated by protein kinase C delta (PKCδ), which is activated through processing of its 78 kDa proform [20,21]. Here, we show significant processing of PKCδ in SCL-I in response to treatment with DKP-071 and DKP-073, and even stronger in response to the combinations with TRAIL, indicated by loss of its pro-form and characteristic cleavage products of 37 and 25 kDa (Figure 4). Additionally, in SCL-II and SCC-12, indications of PKCδ activation by DKP-071 and -073 were visible by induction of the 37 kDa fragment (Figure 5a). However, PKCδ may play an only contributory role here, as indirubin-mediated proapoptotic effects could not be prevented by the PKC inhibitor bisindolylmaleimide I (data not shown).

### 3.5. Regulation of Characteristic Mediators of Apoptosis and Cell Proliferation

To further address the mechanisms of antitumor effects mediated by indirubin derivatives in cSCC cells, the expression of several regulators of apoptosis and cell proliferation was investigated by Western blotting. Thus, pSTAT3 was downregulated in SCL-II and SCC-12 by DKP-071 and -073 (median factor of 2–3, according to densitometric analysis; Figure 5a), indicating an inhibition of the pSTAT3 survival pathway. The TRAIL receptor, DR5, was upregulated by DKP-071 and -073 in SCL-I, SCL-II and SCC-12 (median factors 2–3; Figure 5a,b). The caspase-3 antagonist XIAP (chromosome X-linked inhibitor of apoptosis protein) was downregulated by all three indirubins in SCC-12 and SCL-II as well as by DKP-071 and -073 in SCL-I (factors of 3–10; Figure 5a,b). Additionally, the cIAP survivin appeared as downregulated by the combinations (Figure 5b).

Intrinsic apoptosis pathways are critically controlled by the family of pro- and anti-apoptotic Bcl-2 proteins. Here, we found upregulation of the proapoptotic Bcl-2 protein Puma in SCL-II and SCC-12 (factors of 2–4), whereas antiapoptotic Bcl-2 was downregulated in SCC-12 (factor of 2; Figure 5a). In contrast, no significant changes were obtained for the anti-apoptotic Bcl-2 family members Mcl-1 and Bcl-w or the multidomain proapoptotic family members Bax and Bak (data not shown). Finally, upregulation of the CDK inhibitor p21 was seen in SCL-I in response to indirubin treatment (factor of 4). In course of strongly induced apoptosis after combination treatment, however, it was degraded, as seen by an increased 15 kDa fragment (Figure 5b). Thus, several different pathways appeared as affected by indirubin derivatives in cSCC cells.

### 3.6. Function of Caspases

**Figure 6:**
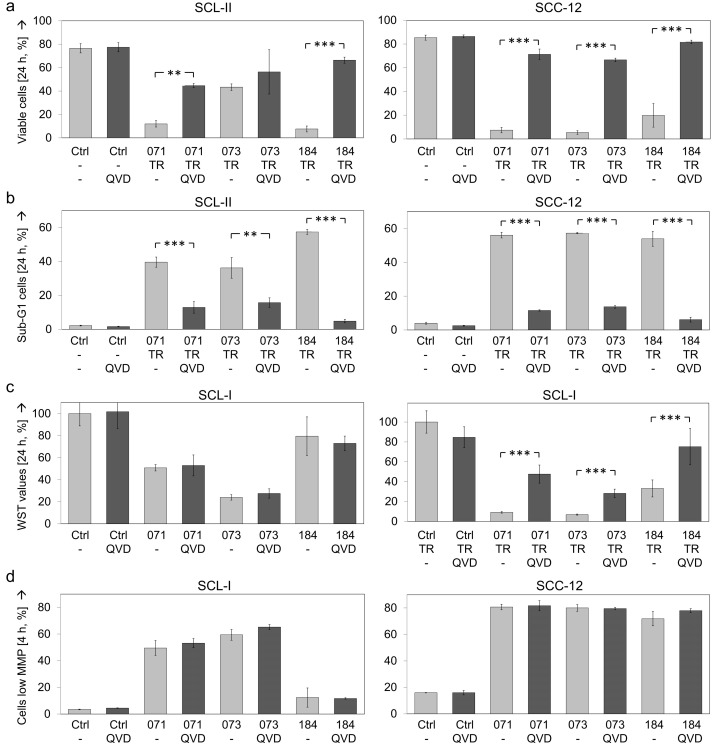

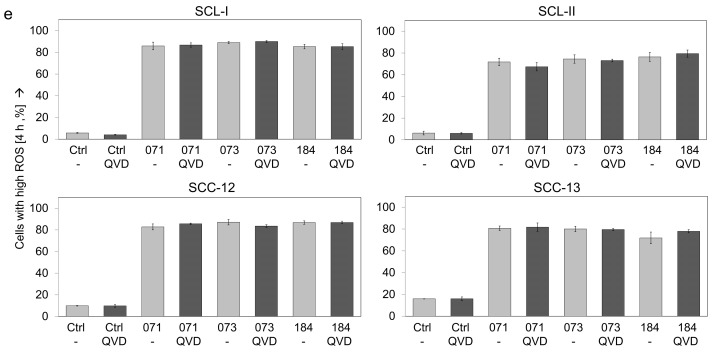
Inhibition of apoptosis by caspase antagonist. The antagonistic effects of the pan-caspase inhibitor QVD-Oph (QVD, 10 µM, 1 h pretreatment) on indirubins’ antitumor activity are shown. (**a**) Cell viability was determined by calcein-AM staining in SCL-II and SCC-12 at 24 h in response to the combinations of indirubins (DKP-071, -073 and -184, 10 μM) and TRAIL (TR; 50 ng/mL). (**b**) Apoptotic cells were quantified by cell cycle analyses (propidium iodide staining, flow cytometry) as sub-G1 cells characterized by DNA fragmentation (treatment as above). (**c**) Cell proliferation was quantified by WST-1 assay in SCL-I at 24 h of treatment with indirubins, TRAIL or combinations. (**d**) Loss of MMP was investigated in SCL-I and SCC-12 at 4 h of treatment with indirubins (10 μM). (**e**) ROS production was investigated in the four cell lines at 4 h of treatment with indirubins (10 μM). (**a**–**e**) Mean values and SDs are shown of a representative experiment (two independent experiments, each one with triplicates). Asterisks indicate statistical significance, when cells ± QVD-Oph were compared (** *p* < 0.01; *** *p* < 0.001).

To prove the significance of caspase activation for the antitumor effects of indirubin derivatives, the pan-caspase inhibitor QVD-Oph was applied. Both decrease of cell viability and induction of apoptosis mediated by indirubin/TRAIL combinations were strongly diminished by QVD-Oph. Thus, in SCL-II, cell viability rates decreased by combinations of TRAIL with DKP-071/073/184 (12%, 42% and 8%) were recovered to 45%, 38% and 66%, respectively. Similarly, in SCC-12, decreased cell viability rates by indirubin/TRAIL combinations (4%, 3% and 13%) were recovered to 65%, 52% and 69%, respectively (Figure 6a).

Comparably, apoptosis that was induced in SCL-II by indirubin/TRAIL combinations (40%, 36%, 57%) was reduced by QVD-Oph to 13%, 16% and 5%, respectively. Similarly, in SCC-12, induced apoptosis by combination treatments (56%, 57% and 54%) was reduced by QVD-Oph to 11%, 14% and 6%, respectively (Figure 6b).

Cell proliferation rates were investigated in SCL-I for indirubin treatment alone and for combinations with TRAIL. Thus, decreased cell proliferation in response to indirubins alone (29–48%) were restored to 35–69%, and decreased cell proliferation in response to indirubin/TRAIL combinations (8–17%) was restored to 36–69% (Figure 6c).

In clear contrast, QVD-Oph pretreatment remained without an effect on loss of MMP, as demonstrated in SCL-I and SCC-12 (Figure 6d), and it remained without an effect on the indirubin-mediated increase of ROS levels, as shown in all 4 cell lines (Figure 6e). Thus, caspases appeared as strongly contributing to induction of apoptosis and loss of cell viability, but they were not upstream of mitochondrial activation and ROS production.

### 3.7. Critical Roles of ROS

**Figure 7:**
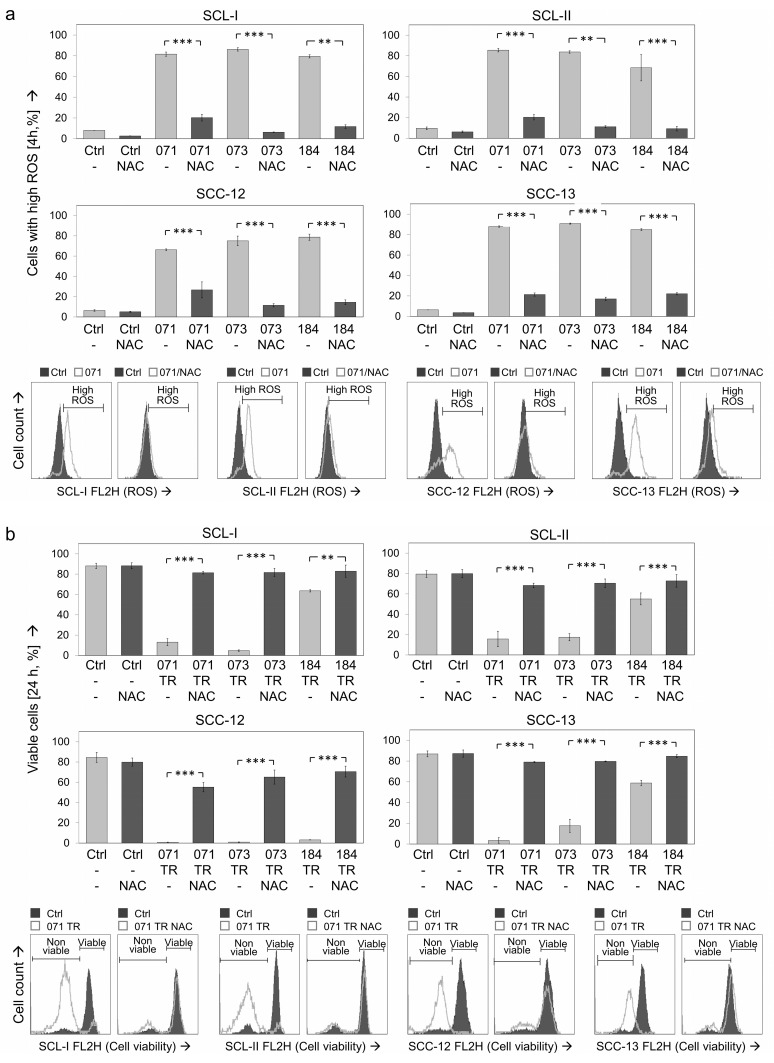
Antagonistic effects of NAC on ROS production and cell viability. The antagonistic effects of the antioxidant N-acetylcysteine (NAC, 1 mM, 1 h pretreatment) were investigated. (**a**) ROS production, as determined by H_2_DCF-DA staining, is shown in the four cSCC cell lines at 4 h of treatment with indirubins (DKP-071, -073 and -184, 10 μM). (**b**) Cell viability was determined by calcein staining in cell lines at 24 h in response to the combinations of indirubins (DKP-071, -073 and -184, 10 μM) and TRAIL (TR; 50 ng/mL). (**a**,**b**) Examples of flow cytometry measurements are provided below (overlays of treated cells vs. controls). Bar charts, mean values and SDs are shown of a representative experiment (two independent experiments, each one with triplicates). Asterisks indicate statistical significance, when cells ± NAC were compared (** *p* < 0.01; *** *p* < 0.001).

To prove the significance of ROS induction for the antitumor effects of indirubin derivatives in cSCC cells, the antioxidant N-acetylcysteine (NAC) was applied. Pretreatment with NAC (1 mM) for 1 h almost completely abolished ROS production in all four cell lines (Figure 7a). This also resulted in almost completely restored cell viability. Thus, cell viability rates that were decreased by combinations of TRAIL with DKP-071 and -073 to values of 4–18% were improved by NAC pretreatment to values >63% (controls at 80–90%) (Figure 7b).

**Figure 8:**
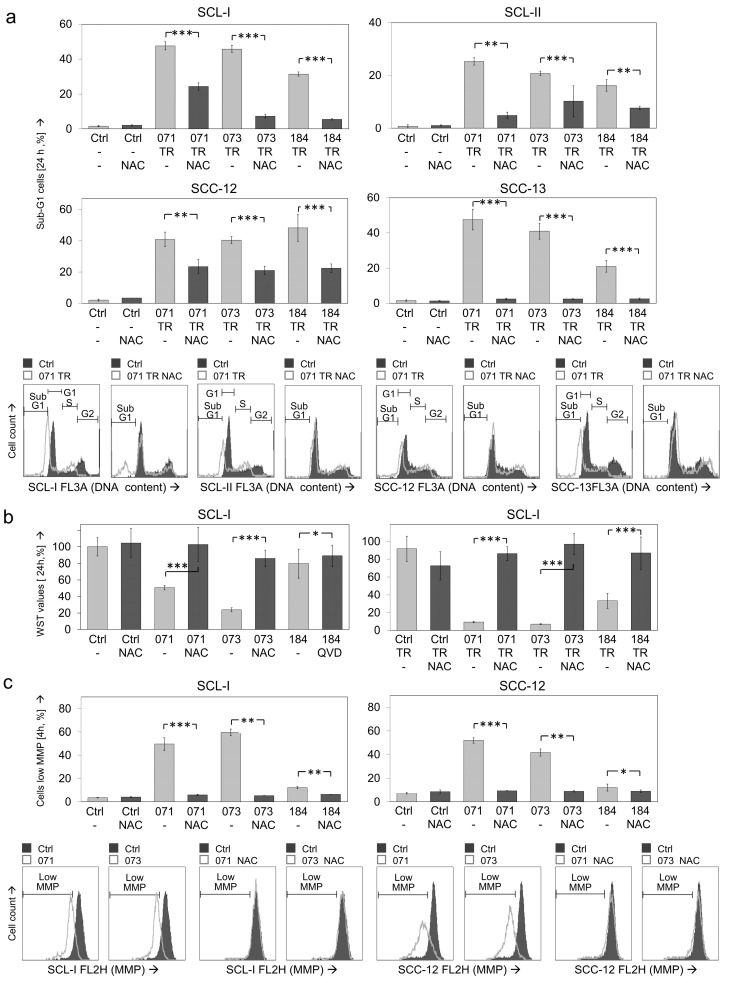
Effects of NAC on apoptosis, cell proliferation and MMP. The antagonistic effects of the antioxidant N-acetylcysteine (NAC, 1 mM, 1 h pretreatment) were investigated. Cells were treated with combinations of indirubins (DKP-071, -073 and -184, 10 μM) and TRAIL (TR; 50 ng/mL) or with indirubins alone, as indicated. (**a**) Apoptosis was determined at 24 h by cell cycle analysis and quantification of sub-G1 cells in four cSCC cell lines. (**b**) Cell proliferation was quantified by WST-1 assay in SCL-I at 24 h. (**c**) Loss of MMP was investigated in SCL-I and SCC-12 at 4 h of treatment. (**a**, **c**) Examples of flow cytometry measurements are provided below (overlays of treated cells vs. controls). (**a**–**c**) Bar charts, mean values and SDs are shown of a representative experiment. Two independent experiments were performed, each one with triplicates. Asterisks indicate statistical significance, when cells ± NAC were compared (* *p* < 0.05; ** *p* < 0.01; *** *p* < 0.001).

Largely comparable findings were obtained at the apoptosis level. Thus, apoptosis induced in SCL-I by combinations of TRAIL with DKP-071/073/184 (48%, 46%, 32%) was reduced by NAC to 24%, 7% and 6%. In SCL-II, apoptosis induced by indirubin/TRAIL combinations (25%, 21%, 16%) was reduced by NAC to 5%, 10% and 8%. Similarly, apoptosis was reduced by NAC in SCC-12 from 41%, 40% and 48% to 23%, 21% and 22%, as well as in SCC-13 from 48%, 41% and 21% to 2%, 2% and 3%, respectively (Figure 8a).

Cell proliferation rates (WST-1 assay) in response to indirubins ± TRAIL were determined in SCL-I. Also here, decreased cell proliferation rates by indirubins alone (29–48%) and in combinations with TRAIL (8–17%) were largely restored to control levels (>78%; Figure 8b). Inhibition of ROS production also strongly affected the loss of MMP. Thus, loss of MMP in SCL-I (50% and 59% by DKP-071/073) and in SCC-12 (63%, 78% of cells) was almost completely abolished by NAC (Figure 8c).

**Figure 9:**
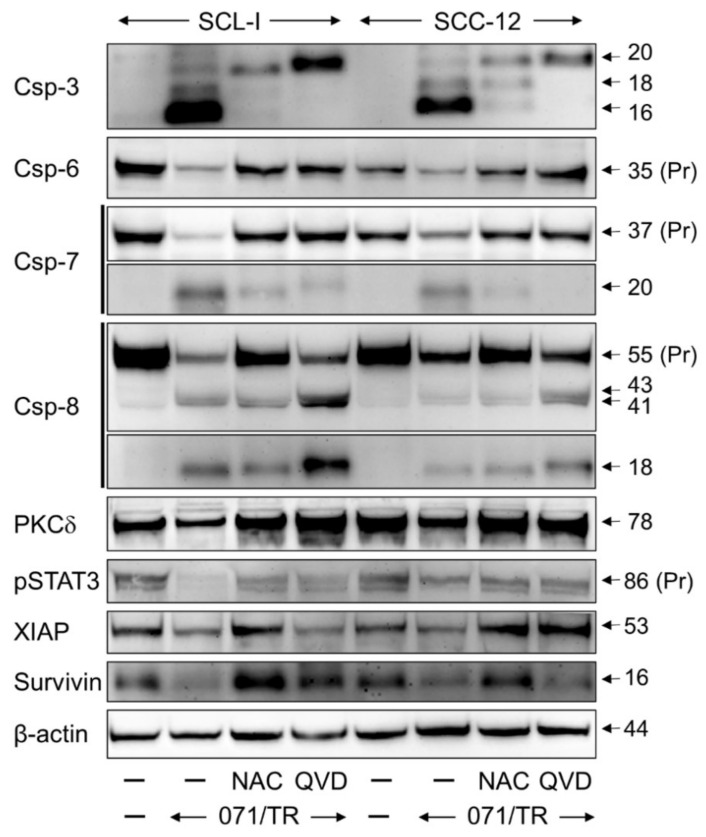
Effects on expression of apoptosis regulators. Expression analysis of characteristic apoptosis regulator proteins in SCL-I and SCC-12 is shown by Western blotting. Cells received pretreatment for 1 h with N-acetylcysteine (NAC, 1 mM) or QVD-Oph (QVD, 10 μM), followed by combination of DKP-071 (071; 10 μM) and TRAIL (TR; 50 ng/mL). Proteins had been extracted at 24 h of treatment. Size of proteins (in kDa) is indicated on the right side, as determined in comparison to a protein size marker run in parallel. Expression of β-actin is shown as loading control. Largely similar results were obtained in three independent Western blot experiments using three independent series of cell extracts.

The antagonistic effects of ROS scavenging through NAC were further investigated in SCL-I and SCC-12 by Western blotting. Thus, processing/activation of effector caspases-3, -6 and -7 by DKP-071/TRAIL treatment was completely abolished by NAC, as it was also abolished by the pan-caspase inhibitor QVD-Oph. Interestingly, caspase-8 processing was also diminished by NAC but not by QVD-Oph (Figure 9), indicating that initiator caspase-8 activation was also downstream of ROS induction.

Additionally, most other activation steps identified in response to indirubin treatment, such as PKCδ and STAT3 activation as well as the downregulation of XIAP and survivin, appeared as downstream of ROS, as they were also reverted by NAC pretreatment (Figure 9). These data clearly favor ROS as the master regulator of indirubin-mediated effects in cSCC cells.

## 4. Discussion

Epithelial skin cancer represents the most common malignancy worldwide. Thus, cutaneous squamous cell carcinoma (cSCC) ranks at second place (~20%) following basal cell carcinoma (~77%) [35]. The incidence of actinic keratosis is even higher, which is defined as carcinoma in situ of cSCC and thus needs treatment [36]. Present standard care of cSCC is mainly based on surgical excision and on chemotherapy. However, frequent side effects and insufficient efficacy of chemotherapy as well as infections by surgery represent major problems [37,38]. With the success of new, targeted therapies, these also gain particular attention for cSCC. Targeted therapy may in particular apply for actinic keratosis, which is often widely distributed but not severe enough for chemotherapeutic treatment.

The natural compound indirubin was identified as an active component of the traditional Chinese medicine Danggui Longhui Wan, used for treatment of chronic and inflammatory diseases [39]. The antitumor activity of indirubin was considered, and clinical trials proved its activity in chronic myeloid leukemia and chronic granulocytic leukemia as well as in head and neck cancer. Importantly, no severe toxicity and side effects have been reported [27,28,40].

Indirubin activity may be further enhanced by chemical modifications, and several new derivatives were described that showed antitumor activities in in vitro and in vivo tumor models, such as for non-small cell lung cancer, glioblastoma, breast, bladder, thyroid, hepatocellular and colorectal cancer [41,42,43,44,45,46]. We have previously reported a series of new indirubin derivatives based on N-glycosylated 3-alkylideneoxindoles containing halogen substituents [30,32]. Two derivatives (DKP-071 and DKP-073) have been previously tested in melanoma and cutaneous T-cell lymphoma cells, where they induced apoptosis in combination with the death ligand TRAIL and affected cell viability [25,47].

In epithelial skin cancer, such as cSCC and basal cell carcinoma, indirubins have not been reported thus far. Here, we show a particularly high efficacy of these derivatives in cSCC cells, which was much stronger as compared to equimolar concentrations of non-substituted indirubin. The active concentration of 10 µM appeared as moderate and may also be reached in tumor tissue.

Indirubin effects were strongly enhanced in combinations with the death ligand TRAIL. TRAIL is a promising candidate for tumor therapy itself, but TRAIL or TRAIL receptor agonistic antibodies have revealed only limited efficacy in clinical trials so far [6]. Thus, the identification of suitable combination partners appears of particular interest. Enhancement of TRAIL-induced apoptosis in cSCC has also previously been shown in combinations with diclofenac and celecoxib [24,33]. As death ligands represent basic elements of an antitumor immune response driven by cytotoxic T-lymphocytes and natural killer cells [6], it is also conceivable that indirubins might enhance an immune response against cSCC in patients. However, first, cytotoxicity studies and investigations of possible side effects on immune cells are needed. Although non-substituted indirubins were quite well-tolerated in clinical trials, no in vivo data are so far available on the compounds investigated here. As many different normal cell types cannot be tested in vitro and as normal cells may reveal largely different characteristics in culture, tolerability of the substances needs to be thoroughly tested in animal models.

The mode of action of indirubins in cancer cells still largely remains in the dark. Inhibition of protein kinases such as JAK, Src, GSK-3β (glycogen synthase kinase), aurora kinase A and cyclin-dependent kinases (CDKs) have been reported [39,48]. Three major types of cell death were distinguished, namely apoptosis, autophagy and necrosis. In principle, apoptosis can be mediated by caspase-dependent or caspase-independent pathways [23]. Intrinsic apoptosis is further characterized by mitochondrial outer membrane permeability as well as by activation of executioner caspases, mainly caspase-3. Here, we saw strong activation of proapoptotic caspase cascades by indirubins alone and even more by combinations with TRAIL. The essential role of caspases was proven by a pan-caspase inhibitor, which almost completely abolished apoptosis induced by indirubins alone as well as by indirubin/TRAIL combinations.

Proapoptotic caspase cascades may be blocked by cIAPs (cellular inhibitor of apoptosis proteins), as XIAP and survivin inhibit effector caspases and caspase-9 [49]. Thus, downregulation of XIAP and survivin seen here in cSCC cells may contribute to the strongly activated caspase cascade. Downregulation of survivin and XIAP by indirubin derivatives has also been seen in breast cancer, melanoma and CTCL cells [25,47,50,51].

There were strong indications that indirubins induce intrinsic apoptosis pathways, and a number of arguments could be collected. Thus, intrinsic apoptosis is characterized by MOMP (mitochondrial outer membrane permeability) and activation of executioner caspases, mainly caspase-3. MOMP leads to loss of the mitochondrial transmembrane potential, associated with cytochrome c release and caspase-9 activation [8]. In a caspase cascade, caspase-3 can activate other effector caspases (-6 and -7) as well as—in a positive feedback loop—caspase-8. Caspase-3 drives DNA fragmentation, e.g., via cleavage of the inhibitor of caspase-activated DNAse (ICAD). Of particular importance for our present findings, intrinsic apoptosis can be initiated by a variety of microenvironmental perturbations, which in particular also include overload of reactive oxygen species (ROS) [8,52]. Loss of MMP in response to indirubins was also seen in melanoma and CTCL cells [25,47]. Intrinsic apoptosis pathways are critically controlled by the family of pro- and anti-apoptotic Bcl-2 proteins [9]. In relation to loss of MMP, we identified the downregulation of antiapoptotic Bcl-2, while proapoptotic Puma was upregulated by indirubins in cSCC cells.

In many cancer cells, persistent activation of STAT3 pathways can promote tumor cell proliferation, survival, angiogenesis and immune evasion. STAT3 activation may be induced via JAK, Src or other tyrosine kinases [51]. Related to its inhibitory activity against JAK, indirubin-induced apoptosis has been related to STAT3 inhibition, as seen in breast cancer, prostate cancer and melanoma cells [47,50]. Downregulation of phosphorylated STAT3 (active) in response to indirubin is also described here in cSCC cells. STAT3 acts as a transcription factor and its inhibition may result in the downregulation of several antiapoptotic factors, including Bcl-2 and survivin [53], which was also seen here in cSCC cells.

A proapoptotic function of PKCδ has been described in response to DNA-damaging agents, UV radiation, phorbol 12-myristate-12-acetate as well as in response to ROS. PKCδ is activated through processing of its 78 kDa proform, which releases a 41 and a 37 kDa fragment [20,21]. In CTCL cells, PKCδ appeared as a master regulator in PEP005-induced apoptosis [54]. Processing of PKCδ in response to indirubins was also shown here by downregulation of the 78 kDa proform and induction of characteristic fragments of 37 and 25 kDa, whereas the 41 kDa fragment was not efficiently detected by the used antibody. PKCδ activation appeared as downstream of caspase-3, as its processing could be inhibited by caspase inhibition. However, PKCδ may have an only contributory role in this setting, as a PKC inhibitor did not prevent the effects of DKPs.

We furthermore found upregulation of TRAIL receptor-1 (DR5) [55], which may contribute to the enhanced TRAIL sensitivity in cSCC cells. Finally, p21 represents a well-known CDK inhibitor [10]. Its upregulation in cSCC cells by indirubins may critically contribute to the inhibition of cell proliferation seen here. Upregulation of TRAIL receptors by indirubins has also been seen in hepatoma, cervical, colon cancer and melanoma cells [47,56,57], whereas upregulation of p21 by indirubin was reported in human laryngeal carcinoma cells [58].

A particular role is described here for reactive oxygen species (ROS). Besides their described functions in tissue damaging and aging [22], reactive oxygen species (ROS) can also mediate proapoptotic signaling in cancer cells and may explain the proapoptotic effects of several anticancer therapies, as demonstrated in skin cancer cells [23,24,25,47]. Concerning the activities of indirubin derivatives, we had previously shown ROS production by indirubins in melanoma and CTCL cells. As shown in the present study, cSCC cells also responded with massive ROS production already at 4 h of indirubin treatment. Thus, ROS production appears as a general mechanism of indirubins in cutaneous cancer cells. Interestingly, not only apoptotic cells but the whole cell population showed increased ROS levels, as demonstrated by flow cytometry. Thus, all tumor cells could be targeted.

Based on our previous data, ROS could act as a signaling molecule in melanoma and CTCL cells by affecting intrinsic, mitochondrial as well as extrinsic, death receptor-mediated apoptosis pathways [25,47]. Additionally, in cSCC cells, indirubin derivatives mediated highly pleiotropic effects, enclosing caspases, mitochondrial membrane potential, PKCδ, STAT3, p21 and Bcl-2 proteins. Our data strongly suggest ROS as the unique master regulator of these pathways in cSCC cells. Indeed, production of reactive oxygen species turned out as upstream of all other identified effects. Namely, the antioxidant NAC completely abolished the proapoptotic effects of indirubins, restored cell viability and cell proliferation as well as prevented all other effects induced by indirubins. ROS production was also reported for other cSCC therapies, such as for chemotherapy, photothermal therapy [59] and photodynamic therapy [60,61].

## 5. Conclusions

In conclusion, these data suggest indirubin derivatives as possibly promising candidates for therapy of epithelial skin cancer, provided that they may also be tolerated in vivo and in the clinical setting. The identification of ROS as particularly responsive for indirubin-mediated antitumor effects opens new perceptions in cSCC therapy. ROS induction should be considered and may also be suitable for early monitoring of therapeutic effects.

## Data Availability

The data presented in this study are available in article and Supplementary Materials.

## Supplementary Materials

The following are available online at https://www.mdpi.com/article/10.3390/antiox10101514/s1. Figure S1: Cytotoxicity in 4 cell lines (a) Exp 1 (b) Exp 2, Figure S2: Dose dependency for DKP-071 / Comparison with non-substituted Indirubin (a) Cell viability (b) Apoptosis, Figure S3: Apoptosis and cell viability in HaCaT cells in response to DKPs, Figure S4: Dose dependency of ROS production for DKP-071 and Figure S5: Time dependency of ROS production for DKP-071 (a) 1–4 h (b) 24 h.
