# Supplementary material for: Crucial Role of Reactive Oxygen Species (ROS) for the Proapoptotic Effects of Indirubin Derivatives in Cutaneous SCC Cells
Source: Antioxidants (Basel). 2021 Sep 24;10(10):1514. doi: 10.3390/antiox10101514 (PMC8532942; doi:10.3390/antiox10101514)
Supplement: Supplementary file 1 [file antioxidants-10-01514-s001.zip › antioxidants-1286966-supplementary.pdf]

Journal : Antioxidants (ISSN 2076-3921)
Manuscript ID: antioxidants-1286966
Title: Crucial role of reactive oxygen species (ROS) for the proapoptotic effects of indirubin derivatives in cutaneous SCC cells
Authors: Jiaqi Zhu, Peter Langer, Claas Ulrich, Jürgen Eberle *

Supplementary figures

Suppl Figure S1a – Cytotoxicity in 4 cell lines (Exp 1)

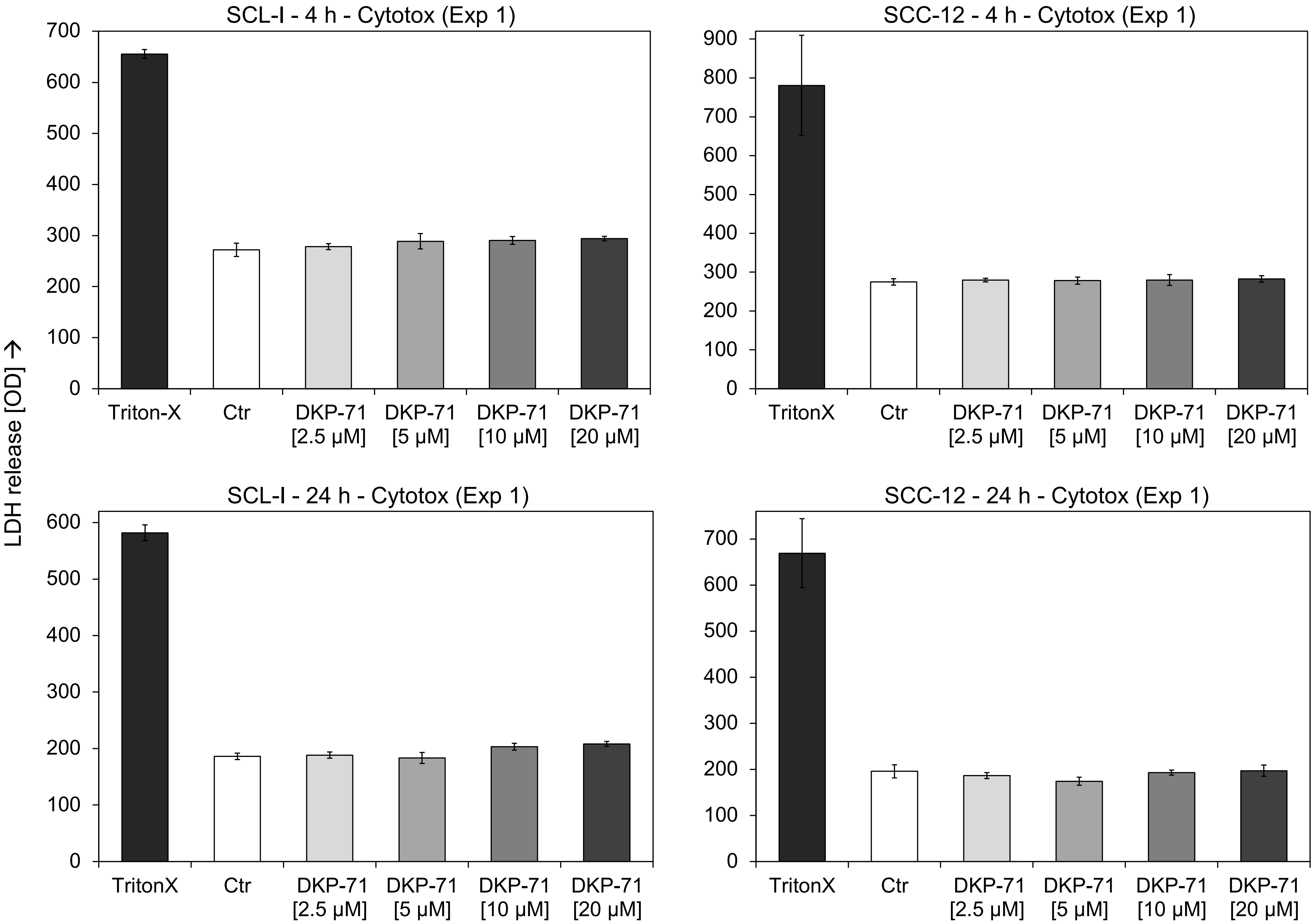

Cell lines SCL-I and SCC-12 were treated with increasing concentrations of DKP-071 (2.5, 5, 10, 20 µM). LDH release assays were performed at 4 h (above) and at 24 h (below). LDH values were compared to non-treated cells (Ctr) as well as to positive controls (cultures lysed with triton X). Experiment 1 of two independent experiments is shown, each one including triplicate values.

Suppl Figure S1b – Cytotoxicity in 4 cell lines (Exp 2)

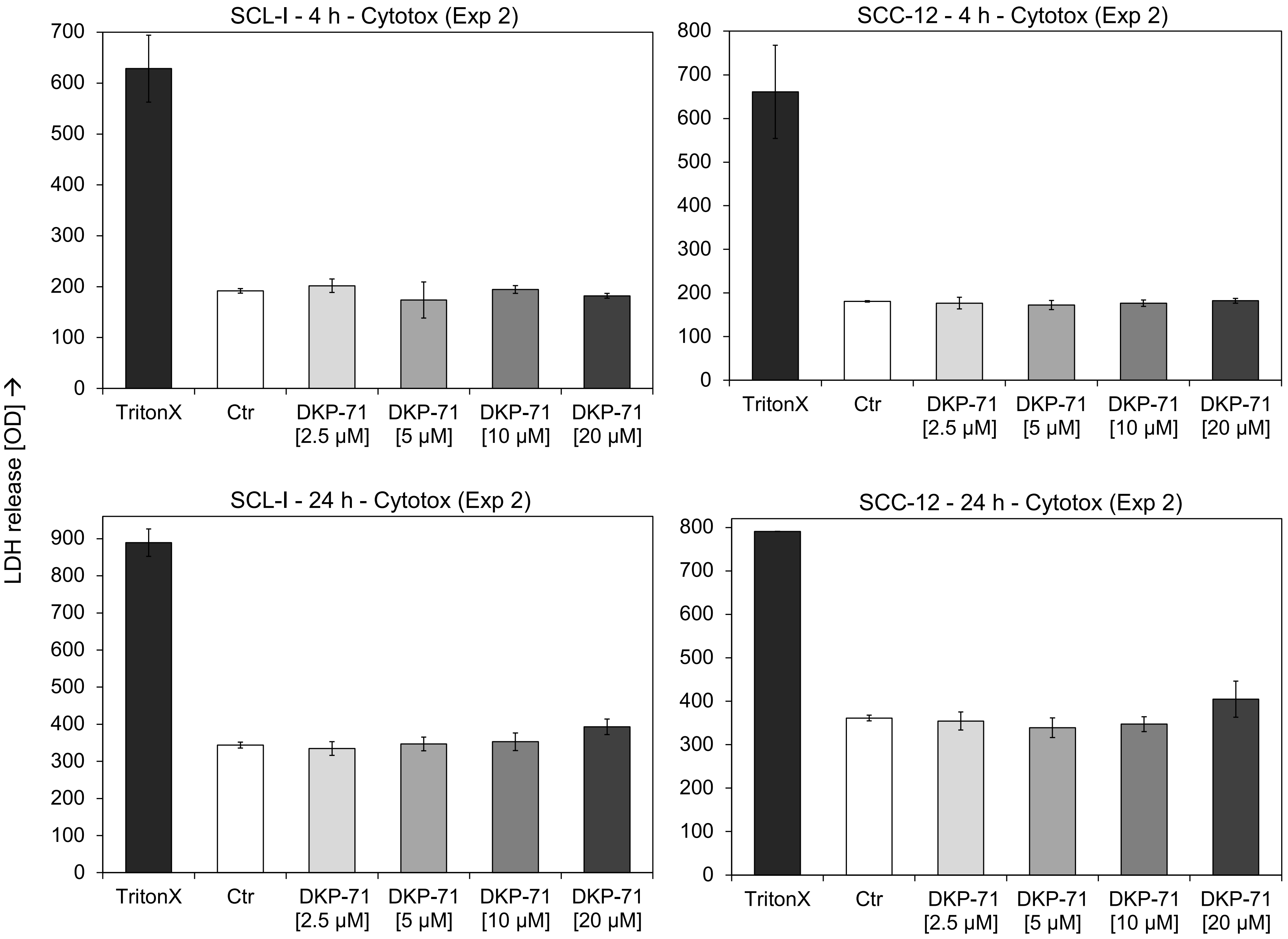

Cell lines SCL-I and SCC-12 were treated with increasing concentrations of DKP-071 (2.5, 5, 10, 20 µM). LDH release assays were performed at 4 h (above) and at 24 h (below). LDH values were compared to non-treated cells (Ctr) as well as to positive controls (cultures lysed with triton X). Experiment 2 of two independent experiments is shown, each one including triplicate values.

Suppl Figure S2a – Dose dependency for DKP-071 / Comparison with non substituted Indirubin (Cell viability)

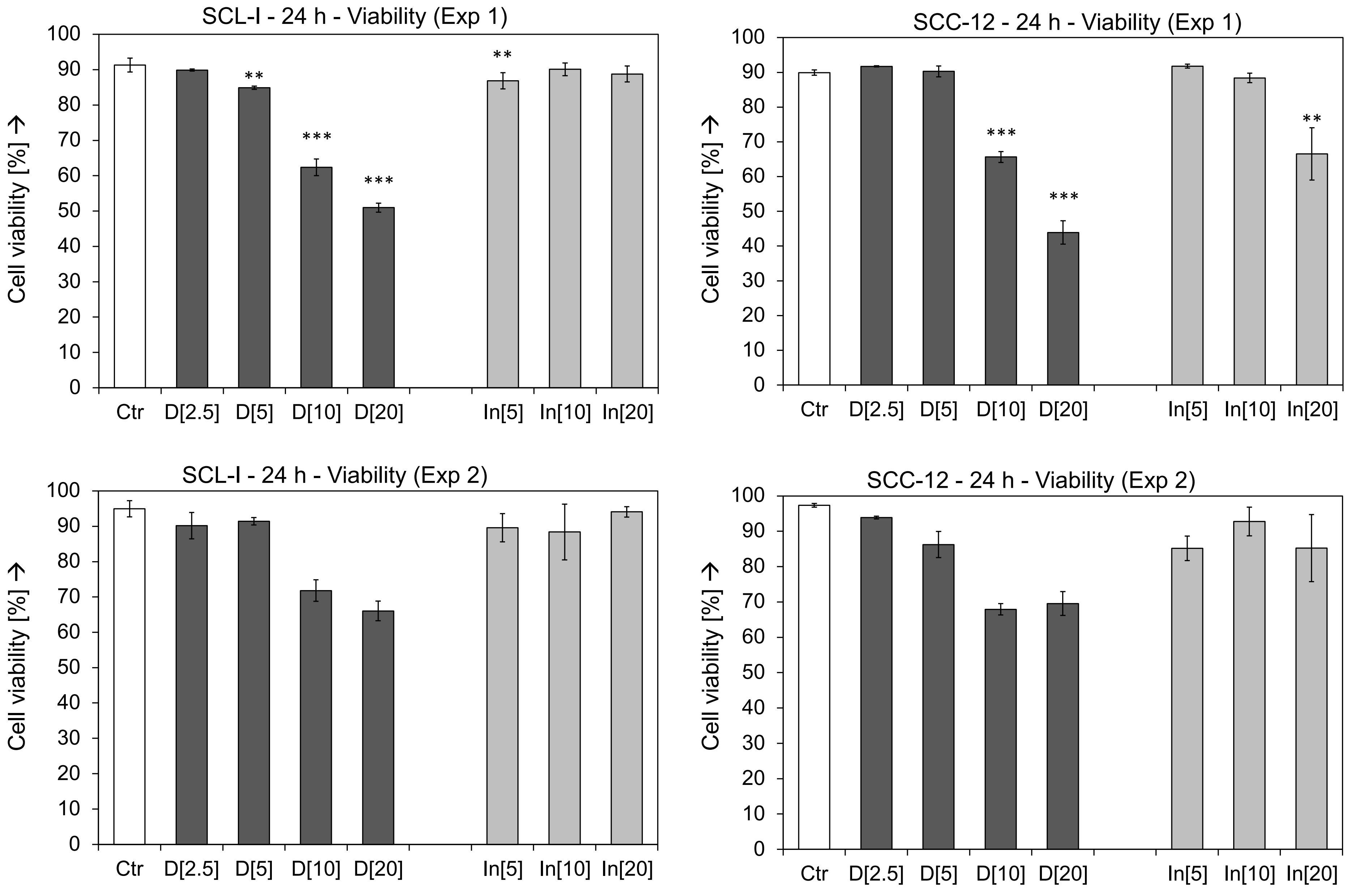

Cell lines SCL-I and SCC-12 were treated with 2.5, 5, 10 and 20 µM of DKP-071 respectively with 5, 10 and 20 µM of non-substituted Indirubin (In). The effects on cell viabilty (Calcein-AM staining) were determined at 24 h of treatment. Ctr, non-treated cells. Two independent experiments are shown for both cell lines (top / bottom), each one including triplicate values. Statistical significance was calculated for all 6 values of both experiments and is indicated for experiment 1 (**, $p < 0.01$; ***, $p < 0.001$).

Suppl Figure S2b – Dose dependency for DKP-071 / Comparison with non substituted Indirubin (Apoptosis)

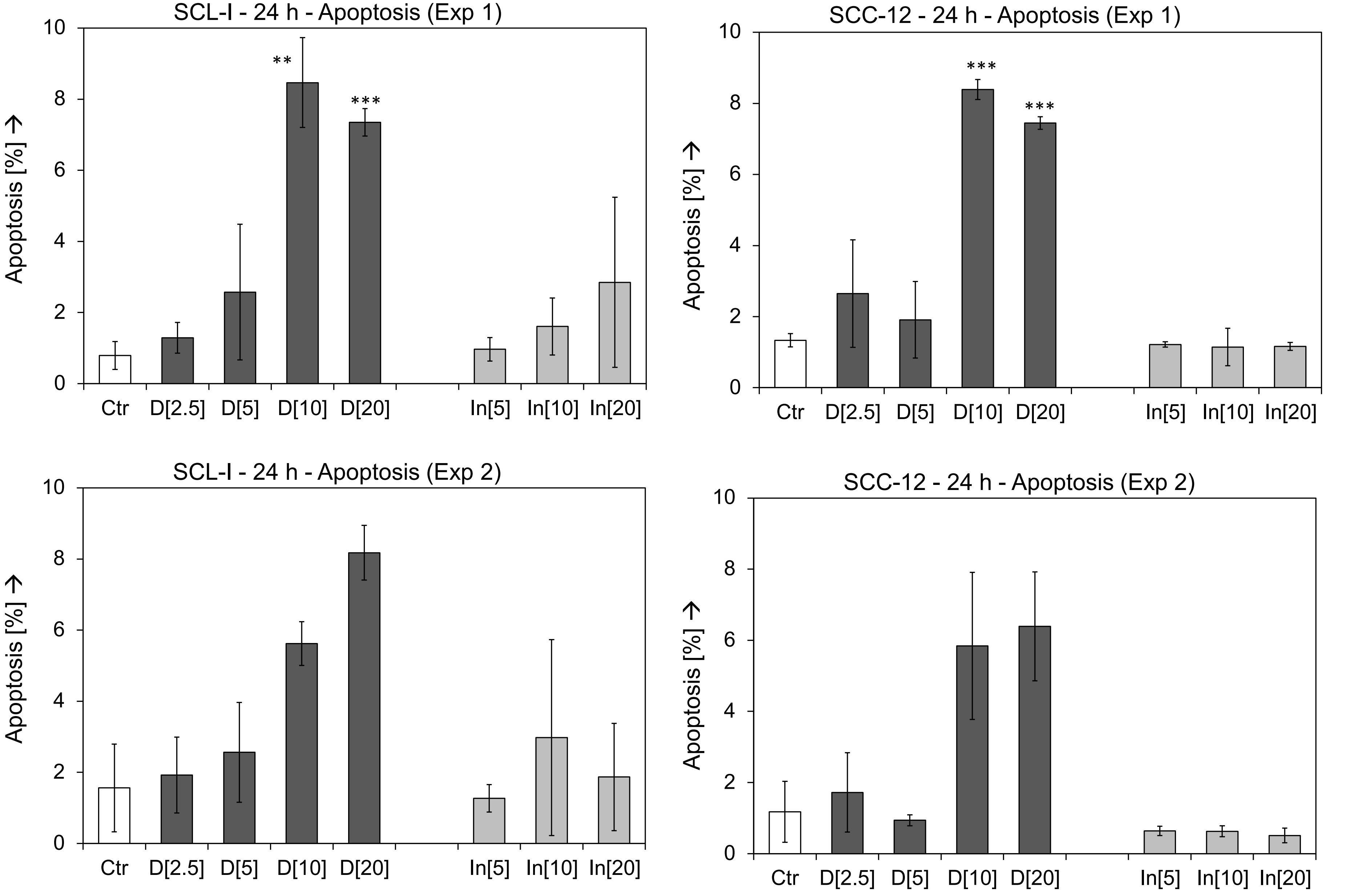

Cell lines SCL-I and SCC-12 were treated with 2.5, 5, 10 and 20 μM of DKP-071 respectively with 5, 10 and 20 μM of non-substituted Indirubin (In). The effects on apoptosis (propidium iodide staining) were determined at 24 h of treatment. Ctr, non-treated cells. Two independent experiments are shown for both cell lines (top / bottom), each one including triplicate values. Statistical significance was calculated for all 6 values of both experiments and is indicated for experiment 1 (**, $p < 0.01$; ***, $p < 0.001$).

Suppl Figire S3 – Apoptosis and cell viability in HaCaT cells in response to DKPs

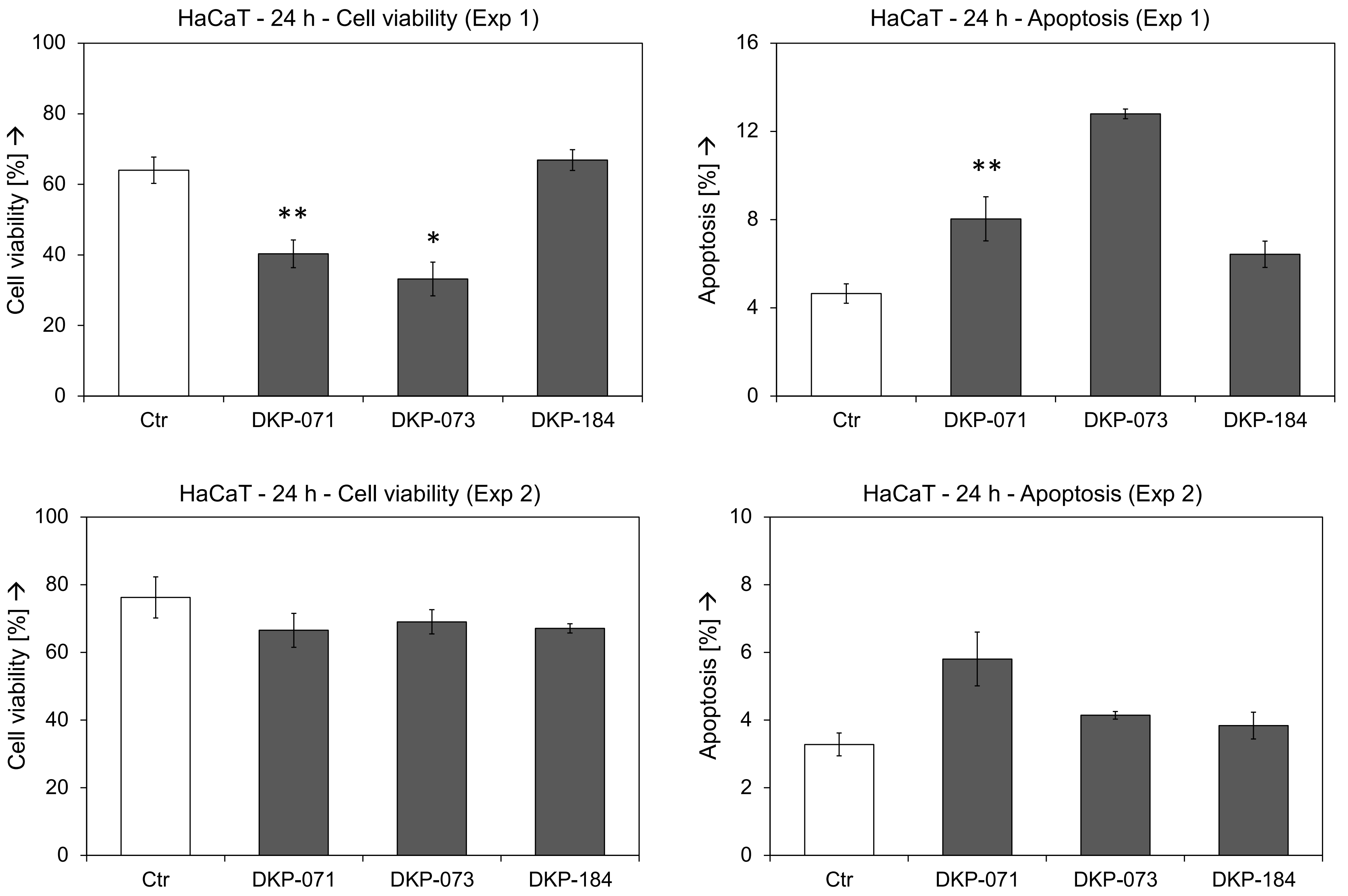

The effects of indirubin derivatives DKP-071, DKP-073 and DKP-184 (10 µM) on cell viability (left) and on apoptosis (right side) were determined in the immortalized keratinocyte cell line HaCaT. Two independent experiments are shown (top / bottom), each one including triplicate values. Statistical significance was calculated for all 6 values of both experiments and is indicated for experiment 1 (*, $p < 0.05$; **, $p < 0.01$).

Suppl Figure S4 – Dose dependency of ROS production for DKP-071

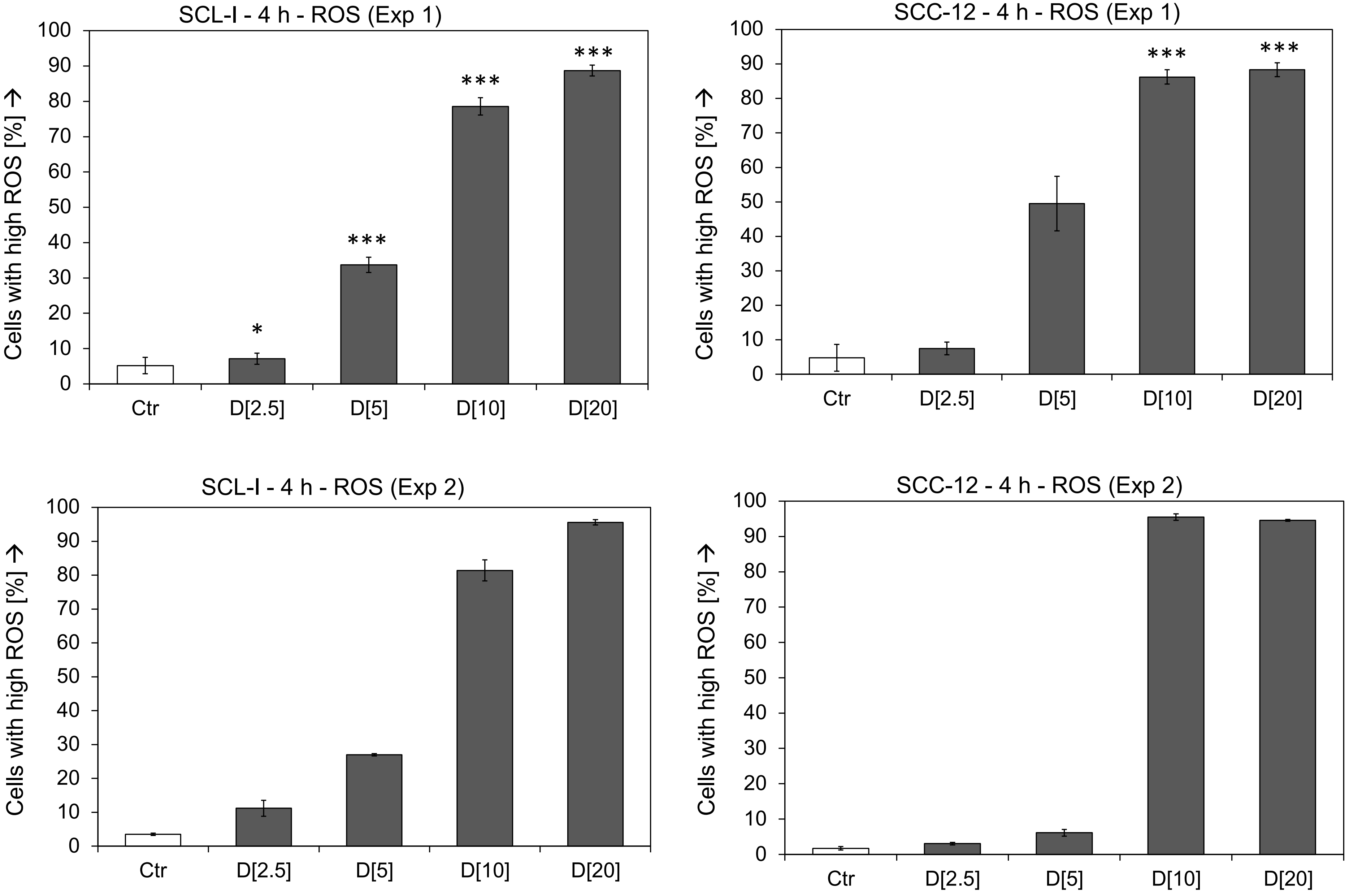

Cell lines SCL-I and SCC-12 were treated with 2.5, 5, 10 and 20 µM of DKP-071. The effects on ROS production were determined at 4 h of treatment (H_2DCF-DA staining). Ctr, non-treated cells. Two independent experiments are shown for both cell lines (top / bottom), each one including triplicate values. Statistical significance was calculated for all 6 values of both experiments and is indicated for experiment 1 (*, $p < 0.05$; ***, $p < 0.001$).

Suppl Figure S5a – Time dependency of ROS production for DKP-071 (1 – 4 h)

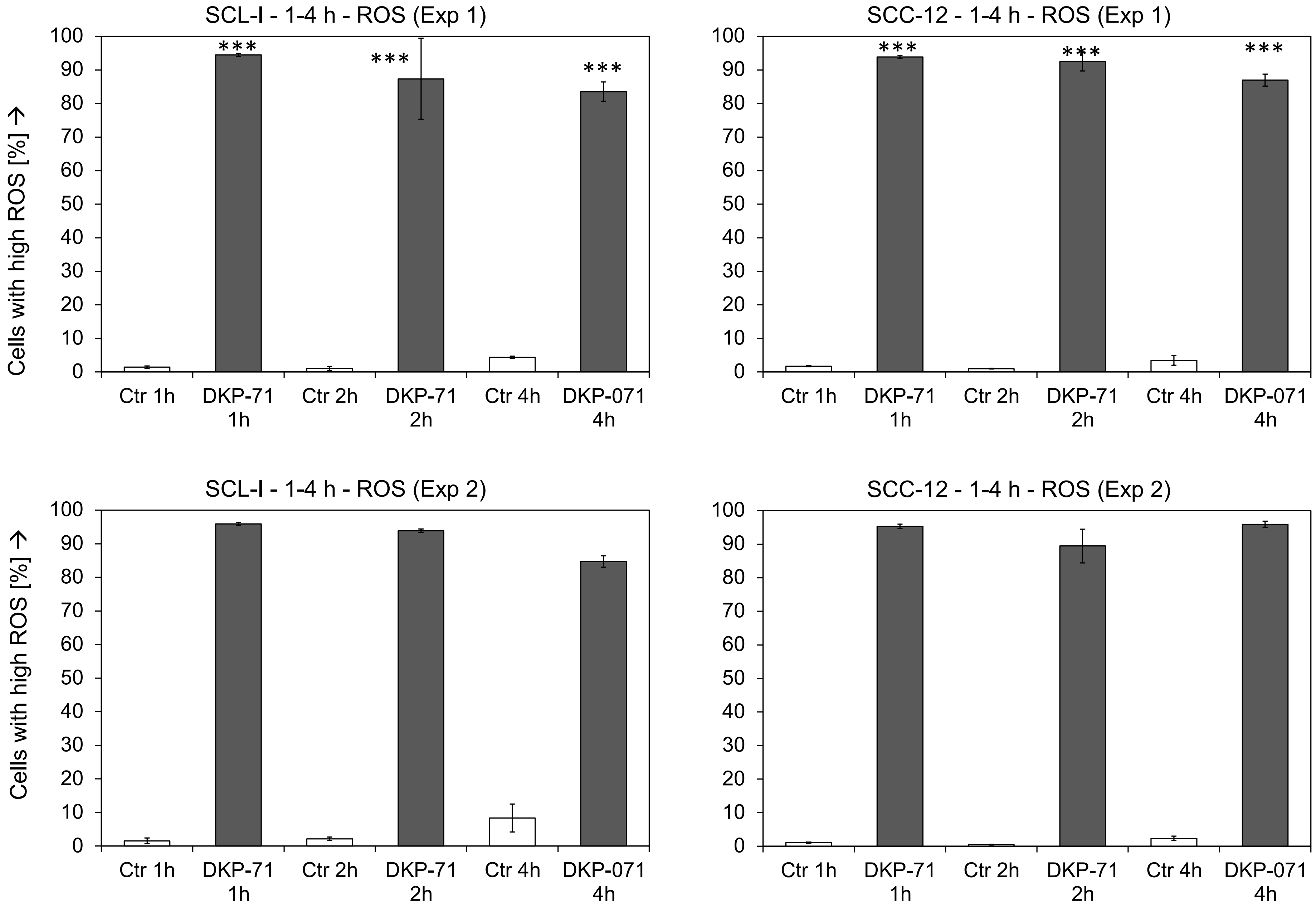

Time dependency of ROS production induced by DKP-071 (10 μM) was determined in SCL-I and SCC-12 at 1 h, 2 h and at 4 h of treatment (H_2DCF-DA staining). Two independent experiments are shown (top / bottom), each one including triplicate values. Statistical significance was calculated for all 6 values of both experiments and is indicated for experiment 1 (***, $p < 0.001$).

Suppl Figure S5b – Time dependency of ROS production for DKP-071 (24 h)

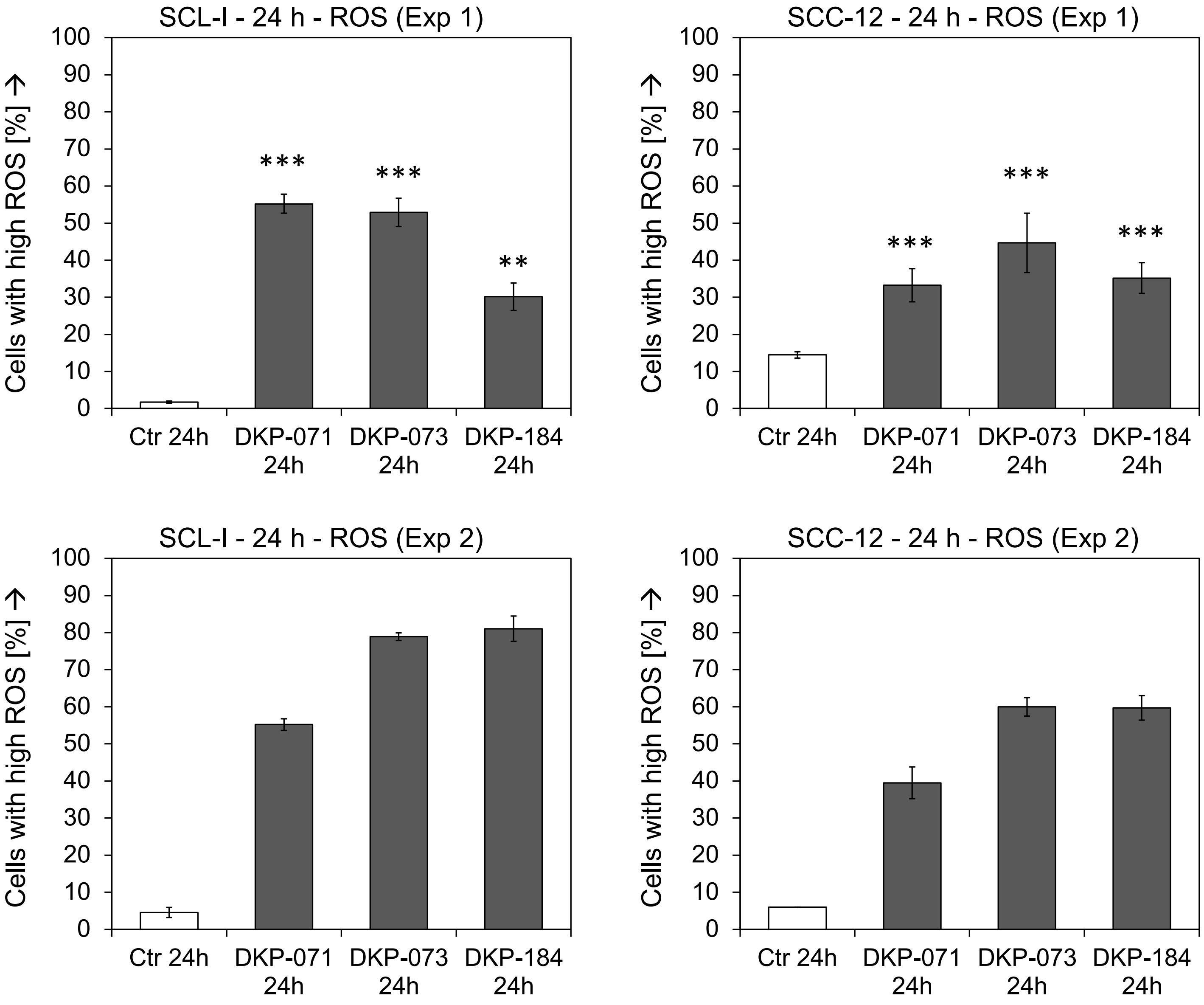

Effects of DKP-071, DKP-073 and DKP-184 (10 µM) on ROS production were determined in SCL-I and SCC-12 at 12 h of treatment (H_2DCF-DA staining). Two independent experiments are shown (top / bottom), each one including triplicate values. Statistical significance was calculated for all 6 values of both experiments and is indicated for experiment 1 (**, $p < 0.01$; ***, $p < 0.001$).
